# Single-dose rAAV5-based vaccine provides long-term protective immunity against SARS-CoV-2 and its variants

**DOI:** 10.1186/s12985-022-01940-w

**Published:** 2022-12-09

**Authors:** Guochao Liao, Hungyan Lau, Zhongqiu Liu, Chinyu Li, Zeping Xu, Xiaoxiao Qi, Yu Zhang, Qian Feng, Runze Li, Xinyu Deng, Yebo Li, Qing Zhu, Sisi Zhu, Hua Zhou, Hudan Pan, Xingxing Fan, Yongchao Li, Dan Li, Liqing Chen, Bixia Ke, Zhe Cong, Qi Lv, Jiangning Liu, Dan Liang, An’an Li, Wenshan Hong, Linlin Bao, Feng Zhou, Hongbin Gao, Shi Liang, Bihong Huang, Miaoli Wu, Chuan Qin, Changwen Ke, Liang Liu

**Affiliations:** 1 Joint Laboratory for Translational Cancer Research of Chinese Medicine of the Ministry of Education of the People’s Republic of China, International Institute for Translational Chinese Medicine, Guangzhou University of Chinese Medicine, Guangzhou, China; 2 State Key Laboratory of Dampness Syndrome of Chinese Medicine, The Second Affiliated Hospital of Guangzhou, University of Chinese Medicine, Guangzhou, China; 3 Guangzhou Laboratory, Guangzhou, China; 4 Guangdong Hengda Biomedical Technology Co., Ltd., Guangzhou, China; 5 Guangdong Provincial Center for Disease Control and Prevention, Guangzhou, China; 6 National Human Diseases Animal Model Resources Center, Key Laboratory of Human Disease Comparative Medicine, Chinese Ministry of Health, Beijing Key Laboratory for Animal Models of Emerging and Reemerging Infectious Diseases, Institute of Laboratory Animal Science, Chinese Academy of Medical Sciences and Comparative Medicine Center, Peking Union Medical College, Beijing, China; 7 Guangdong Keguanda Pharmaceutical Technology Co., Ltd., Guangzhou, China; 8 State Key Laboratory of Quality Research in Chinese Medicine, Macau University of Science and Technology, Macao, Macao SAR China; 9 Queen Mary Hospital; LKS Faculty of Medicine, The University of Hong Kong, Hong Kong, China; 10 Guangdong Provincial Key Laboratory of Laboratory Animals, Guangdong Laboratory Animals Monitoring Institute, Guangzhou, China

**Keywords:** SARS-CoV-2, Vaccine, rAAV5, Neutralizing antibodies, Virus challenge

## Abstract

The COVID-19 pandemic, caused by the SARS-CoV-2 virus and its variants, has posed unprecedented challenges worldwide. Existing vaccines have limited effectiveness against SARS-CoV-2 variants. Therefore, novel vaccines to match mutated viral lineages by providing long-term protective immunity are urgently needed. We designed a recombinant adeno-associated virus 5 (rAAV5)-based vaccine (rAAV-COVID-19) by using the SARS-CoV-2 spike protein receptor binding domain (RBD-plus) sequence with both single-stranded (ssAAV5) and self-complementary (scAAV5) delivery vectors and found that it provides excellent protection from SARS-CoV-2 infection. A single-dose vaccination in mice induced a robust immune response; induced neutralizing antibody (NA) titers were maintained at a peak level of over 1:1024 more than a year post-injection and were accompanied by functional T-cell responses. Importantly, both ssAAV- and scAAV-based RBD-plus vaccines produced high levels of serum NAs against the circulating SARS-CoV-2 variants, including Alpha, Beta, Gamma and Delta. A SARS-CoV-2 virus challenge showed that the ssAAV5-RBD-plus vaccine protected both young and old mice from SARS-CoV-2 infection in the upper and lower respiratory tracts. Whole genome sequencing demonstrated that AAV vector DNA sequences were not found in the genomes of vaccinated mice one year after vaccination, demonstrating vaccine safety. These results suggest that the rAAV5-based vaccine is safe and effective against SARS-CoV-2 and several variants as it provides long-term protective immunity. This novel vaccine has a significant potential for development into a human prophylactic vaccination to help end the global pandemic.

## Introduction

As of May 23, 2022, severe acute respiratory syndrome coronavirus-2 (SARS-CoV-2) has caused more than 522 million infections and 6.3 million deaths. The SARS-COV-2 pandemic has provided many opportunities for mutation of the single-stranded RNA viral genome. More than 1000 novel variants have been detected worldwide, among which the World Health Organization (WHO) has identified five mutant strains of concern [1, 2]. In particular, the circulating sub-variants of Delta and Omicron resulted in an accelerated worldwide spread. Various studies have shown the Delta variant to be more infectious and dangerous, reducing the efficacy and protection of existing vaccines by inducing immune escape, leading to re-infection in people who have recovered from wild-type SARS-CoV-2 [3–5]. Currently, there are more than 150 vaccines in clinical trials and nearly 200 pre-clinical vaccine candidates [6, 7]; however, few have shown significant protective efficacy against both SARS-CoV-2 and its circulating variants over the long term, Delta and Omicron variants in particular [5, 8]. Therefore, a vaccine effective against wild-type SARS-CoV-2 and its variants is urgently needed.

Adeno-associated virus (AAV) has been widely used for gene therapy due to its safety and characteristics of high efficacy and low immunogenicity [9–11], which makes it an ideal vaccine vector. The prevalence of pre-existing neutralizing antibodies (NAs) against AAV vectors is directly related to vaccine efficacy [12]. AAV type 5 (AAV5), with low levels of pre-existing NAs (3.2%) [13, 14] and high affinity for human airway epithelia [15], has the potential to be optimized as a vaccine vector, especially since the safety of AAV5 in humans has been well studied and evaluated [13]. A recombinant AAV vector has been effectively used for gene delivery [16, 17], demonstrating its utility in non-human primates. However, the natural rAAV genome is single-stranded DNA (ssDNA) with the limitation of relying on cell replicators to synthesize complementary strands. In our study, we not only initiated the development a series of ssAAV-vectored COVID19 vaccine candidates, but also designed and constructed self-complementation AAV (scAAV) based SARS-CoV-2 vaccines.

Here, we used the AAV5 delivery vehicle to produce a broad vaccine against SARS-CoV-2 and its variants that is protective over the long term in mice. Intramuscular administration of ssAAV5-based or scAAV5-based vaccines elicited robust systemic humoral and cell-mediated immune responses in mice and Wistar rats. Only one single dose of the scAAV5-RBD1 vaccine exhibited adequate antibody titers in mice and Wistar rats until one year. Importantly, the scAAV5-RBD1 vaccine also exhibited a wide range of neutralizing antibody responses to SARS-CoV-2 variants Alpha, Beta and Delta.

## Materials and methods

### Viruses and cells

Vero E6 cells and HEK293 cells (American Type Culture Collection, ATCC) were cultured at 37 °C in Dulbecco's Modified Eagle medium (DMEM) supplemented with 10% fetal bovine serum (FBS), 10 mM HEPES pH 7.4, 1 mM sodium pyruvate, 1 × non-essential amino acids, and 100 U/mL of penicillin-streptomycin as our previously studies [18, 19]. SARS-CoV-2 strain 2019n-CoV/USA_WA1/2020 and SARS-CoV-2 variants, including Alpha, Beta, Gamma and Delta were obtained from the Guangdong Provincial Center for Disease Control and Prevention and Institute of Medical Laboratory Animals of Chinese Academy of Medical Sciences. All experiments with infectious SARS-CoV-2 were performed in BSL3 facilities approved by Institutional Biosafety Committee.

### Animals in the experiments

Six weeks-old, 36 weeks-old female BALB/c mice and 6–8 weeks female Wistar rats were purchased from the Laboratory Animal Center of Southern Medical University (Guangdong, China). The Animal Care and Use Committee at Guangzhou University of Chinese Medicine approved the research. All animals were given a commercial mouse food and water ad libitum and housed in a temperature-controlled environment with a 12-h light-dark cycle.

All animals were immunized with rAAV5-based vaccines or rAAV5-GFP (control group) *via* intramuscular (IM) injection in the hind leg or intranasal inoculation at a single dose (five to eight animals for each group). Sera were collected for cytokines analyses performed by the Macau University of Science and Technology using a Non-Human Primate Cytokine Panel kit (Merck Millipore, Billerica, USA, Cat# PCYTMG-40K-PX23) on a Bio-Plex 200 instrument (Bio-Rad, Hercules, CA, USA) according to the manufacturer’s protocol. For the virus attacking test, mice were challenged on day 40 after immunization with 3.6Log PFU of SARS-CoV-2 (HRB26M strain) *via* intranasal route. Animals were euthanized, and tissues were harvested for further analysis.

### Construction and titration of rAAV5-based vaccines

The recombinant type 5 adenoviral-vectored SARS-CoV-2 (Accession Number: MN985325.1) vaccines encoding RBD domain (residues 319-541), RBD-plus domain (residues 319-583), S1 protein (residues 14-685), full-length of S protein (residues 14-1213) and NTD domain (residues 14-305 of the S protein N-terminal domain) of SARS-CoV-2 were produced by PackGene Biotech (Guangzhou, China). Briefly, rAAV5 packaging plasmids were transfected into HEK293T cells using PEI transfection reagent, according to the manufacturer’s protocol. The transfected cells and supernatants were harvested 72 h post transfection. rAAV5-vaccine was purified and titrated by real-time quantitative PCR. rAAV5-vaccine was adjusted to 10^12^ GCs/mL in PBS and used for the following vaccinations.

### ELISA

Specific IgG and IgM against COVID-19 in mouse sera were tested by ELISA. Briefly, serially diluted mouse sera were added to 96-well microtiter plates pre-coated with RBD-His or S1-His protein. The plates were incubated at 37 °C for 30 min, followed by four washes with PBS containing 0.1% Tween 20 (PBST). Bound Abs were then reacted with HRP-conjugated goat anti-mouse IgG or IgM (Southern Biotech, Birmingham, USA, Cat.#1030-05) at 37 °C for 20 min. After four washes, the substrate 3,3,5,5-tetramethylbenzidine (Southern Biotech, Cat.#1030-05) was added to the plates, and the reaction was stopped by adding 1 N H_2_SO_4_. The absorbance at 450 nm was measured by an ELISA plate reader (Bio-Rad, Hercules, CA, USA). The endpoint serum dilution was calculated with a curve to fit the analysis of optical density (OD) values for serially diluted sera with a cut-off value of negative control.

### Neutralization assay

Titers of NA in sera of mice immunized with rAAV5-GFP or rAAV5-vaccines were detected in Vero E6 cells as in our previous study [18]. Vero E6 cells were seeded at 1×10^4^/well in 96-well culture plates and cultured at 37 °C to form a monolayer. Serial 4-fold dilutions of serum samples were mixed separately with 100 TCID50 (50% tissue-culture infectious dose) of SARS-CoV-2 strain (Guangdong Provincial Center for Disease Control and Prevention and institute of medical laboratory animals of Chinese Academy of Medical Sciences), incubated at 37 °C for 1 h, and added to the monolayer of Vero E6 cells in tetrad. Cells infected with or without 100 TCID_50_ SARS-CoV-2 were applied as positive and negative controls. Each well's cytopathic effect (CPE) was observed daily and recorded on day 3 post infection. The neutralizing titers of mouse antisera that completely prevented CPE in 50% of the wells were calculated by the Reed-Muench method.

### Quantitative RT-PCR

The viral RNA copies in nasal or lung tissues of challenged mice were determined by Quantitative RT-PCR according to the protocol. Total RNA was extracted from 20 mg of lung tissues using a RNeasy Mini kit (Qiagen, Hilden, Germany, Cat.#74104). Then cDNA was synthesized using random primers and the SuperScript II RT kit (Invitrogen, Waltham, MA, USA, Cat.#18064014). Extracted RNA (10 µL) was reverse transcribed in a 20-µL reaction mixture containing 1 × first strand buffer, 100 mM DTT, 10 mM each dNTP, 50 ng of random primers, 40 U of RNaseOUT, and 200 U of SuperScript II RT at 42 °C for 50 min, followed by 15 min at 70 °C. The solution was incubated with RNase H (Invitrogen, Waltham, MA, USA, Cat.# 18021071) at 37 °C for 20 min. Synthesized cDNA was quantified using Power SYBR Green PCR Master Mix (Life Technologies, Carlsbad, California, United States, Cat.#4309155) in a 20-µL mixture containing 5 µL of cDNA (1/10), 10 µL of 2 × Power SYBR Green PCR Master Mix, 3 µL of RNase-free H_2_O, 10 µM forward primer and reverse primer in a Mx3000 QPCR System (Agilent, Santa Clara, California, USA).

### Cell surface markers/intracellular cytokines staining

Single-cell suspensions (3 × 10^6^) from spleens of the vaccinated mice were harvested and stimulated with or without SARS-CoV-2 S-specific peptide (S full-length peptide, residues 1-1213, 1 µg/mL) plus anti-mouse IL-2 (20 U/mL). Cells with stimulatory agents were incubated for 72 h at 37 °C with 5% CO_2_. The cells were harvested and stained directly with conjugated mAbs specific for cell surface markers including CD45, CD3, CD4, CD8, CD44, CD62L (BioLegend, San Diego, USA) for 30 min at 4 °C. Intracellular antigens including IFN-γ, IL-4 and TNF-α were stained for 20 min in the dark at room temperature. The stained cells were analyzed using a flow cytometer (BD Biosciences, San Jose, CA, USA). Data were analyzed by Cell Quest software (BD Biosciences, San Jose, CA, USA).

### Statistical analysis

Statistical significance among different vaccination groups was calculated by the student t test using statistical software (GraphPad Prism 8). Values were presented as mean with SEM. Values of *P* < 0.05 were considered significant.

## Results

### ssAAV5-based vaccine encoding SARS-CoV-2 RBD-plus elicited robustest humoral-mediated immune responses against SARS-CoV-2

**Fig. 1:**
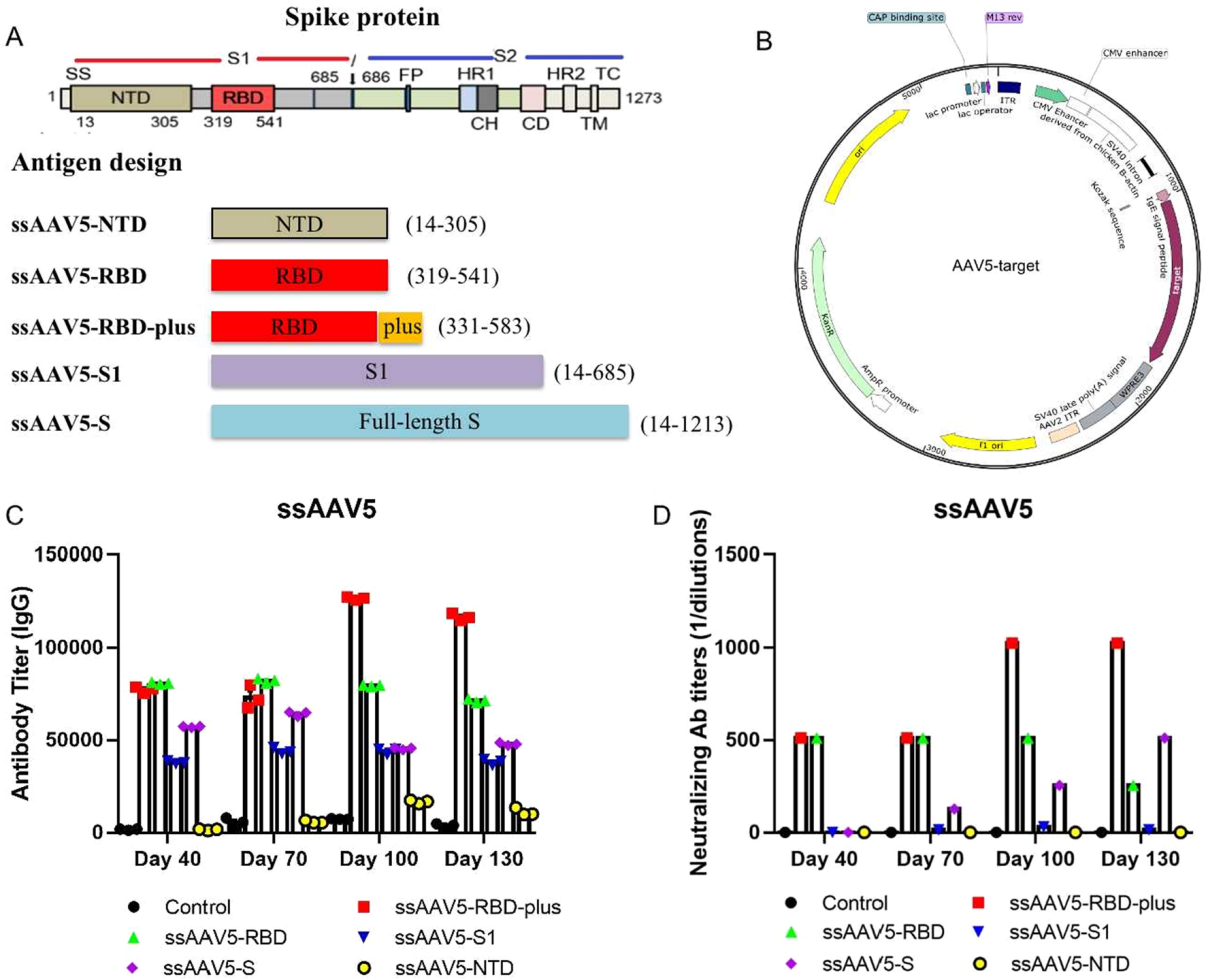
Construction of recombinant AAV5-based vaccines and their immunogenicity. **A** The encoding domains of five recombinant ssAAV5-based vaccines; **B** The experimental design of each AAV-vaccine vector. The vector contains a promoter, IgE secretory signal peptide, various antigen, and ploy A that flanked with two ITR elements. **C** IgG and **D** Wild-type virus-specific NAs were evaluated at 40 days and continued until 130 days after a single-dose immunization. The ssAAV5-RBD-plus vaccine induced the most robust immune responses.

In order to systematically screen for the most effective SARS-CoV-2 spike (S) protein antigens for inducing an immune response, we designed five ssAAV5 vaccines based on the receptor binding domain (RBD) and other S protein regions: ssAAV5-RBD, ssAAV5-RBD-plus, ssAAV5-S1, ssAAV5-S, and ssAAV5-NTD (Fig. 1A). Given the sequence conservation of different S protein domains, we extended the length of antigen sequence in the RBD-plus domain to compare with that of the RBD domain. The immunogenicity of the newly designed vaccines was then assessed in BALB/c mice. Animals were immunized by intramuscular (IM) inoculation with 1 × 10^11^ genome copies (GCs) of each vaccine or a parallel control (AAV5-GFP; 1 × 10^12^ GCs/mL, 100 µL). Antigen-specific humoral immune responses of immunoglobulin G (IgG) (Fig. 1B) and NA titers in wild-type SARS-CoV-2-infected Vero E6 cells were evaluated on days 40–130 after single-dose immunization (Fig. 1C). Among the vaccine groups, ssAAV5-RBD-plus elicited the highest specific IgG and NA titers, indicating that, compared to other antigens, RBD-plus domain residues 331–583 elicited the most robust immune response against wild-type SARS-CoV-2.

### ssAAV5-RBD-plus at the concentration of 1 × 10^11^ exhibited the best cell-mediated immune antigenicity

**Fig. 2:**
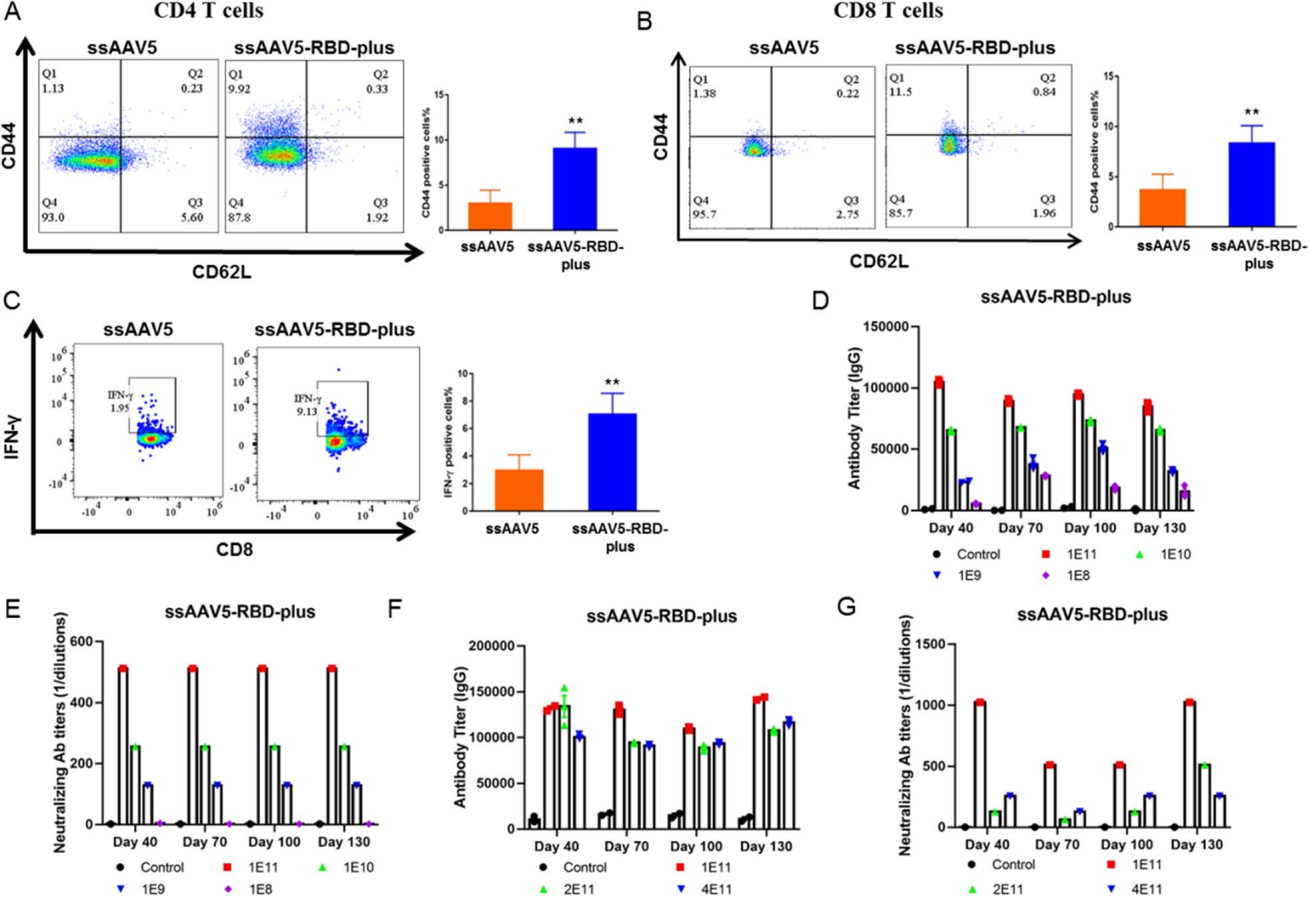
Determination of T-cell immune responses and effective vaccine dose. **A** After 130 days post-immunization with the ssAAV5-RBD-plus vaccine, populations of memory CD4^+^ T and CD8^+^ cells **B** were significantly increased; **C** Secretion of IFN-γ from CD8^+^ cells was significantly upregulated; Among doses of 1×10^11^, 1×10^10^1, 1×10^9^, 1×10^8^ GCs, the amount of IgG (**D**) and NAs (**E**) indicates that 1×10^11^ GCs had the most robust immunogenicity; Among doses of 1×10^11^, 2×10^11^ and 4×10^11^ GCs, the amount of IgG (**F**) and NAs (**G**) 1×10^11^ GCs had the most robust immunogenicity. Values represent mean ± SEM; significant difference versus control group, **P < 0.01.

Next, T-cell responses after vaccination were evaluated in mice. At 130 days post-immunization with the ssAAV5-RBD-plus vaccine, memory CD4^+^ and CD8^+^ T-cells were significantly increased (Fig. 2A, B). Cytokine IFN-γ secretion from CD8^+^ cells was significantly upregulated compared to control animals (Fig. 2C). These results indicate that the ssAAV5-based RBD-plus vaccine can effectively induce robust systemic humoral and cell-mediated immune responses. Optimal dosage is a critical factor for vaccine efficacy. To determine the optimal dosages of the ssAAV5-RBD-plus vaccine, we first vaccinated mice by IM at doses of 1×10^8^, 1×10^9^, 1×10^10^, and 1×10^11^ GCs, respectively. The results showed that titers of IgG and NA were elevated with increasing doses, with animals vaccinated with 1×10^11^ GCs showing the strongest immunogenicity (Fig. 2D, E). To clarify that 1×10^11^ GCs was the most effective dose, higher doses of 2×10^11^ and 4×10^11^GCs were used. As shown in Fig. 2F and G, mice vaccinated with 1×10^11^ GCs achieved the most robust immune responses. Based on these results, 1×10^11^ GCs of the ssAAV5-based vaccine were determined to be the optimal dose and used for further studies.

### A single dose of ssAAV5-RBD-plus vaccine provided substantial protection against wild-type SARS-CoV-2 in old and young mice

**Fig. 3:**
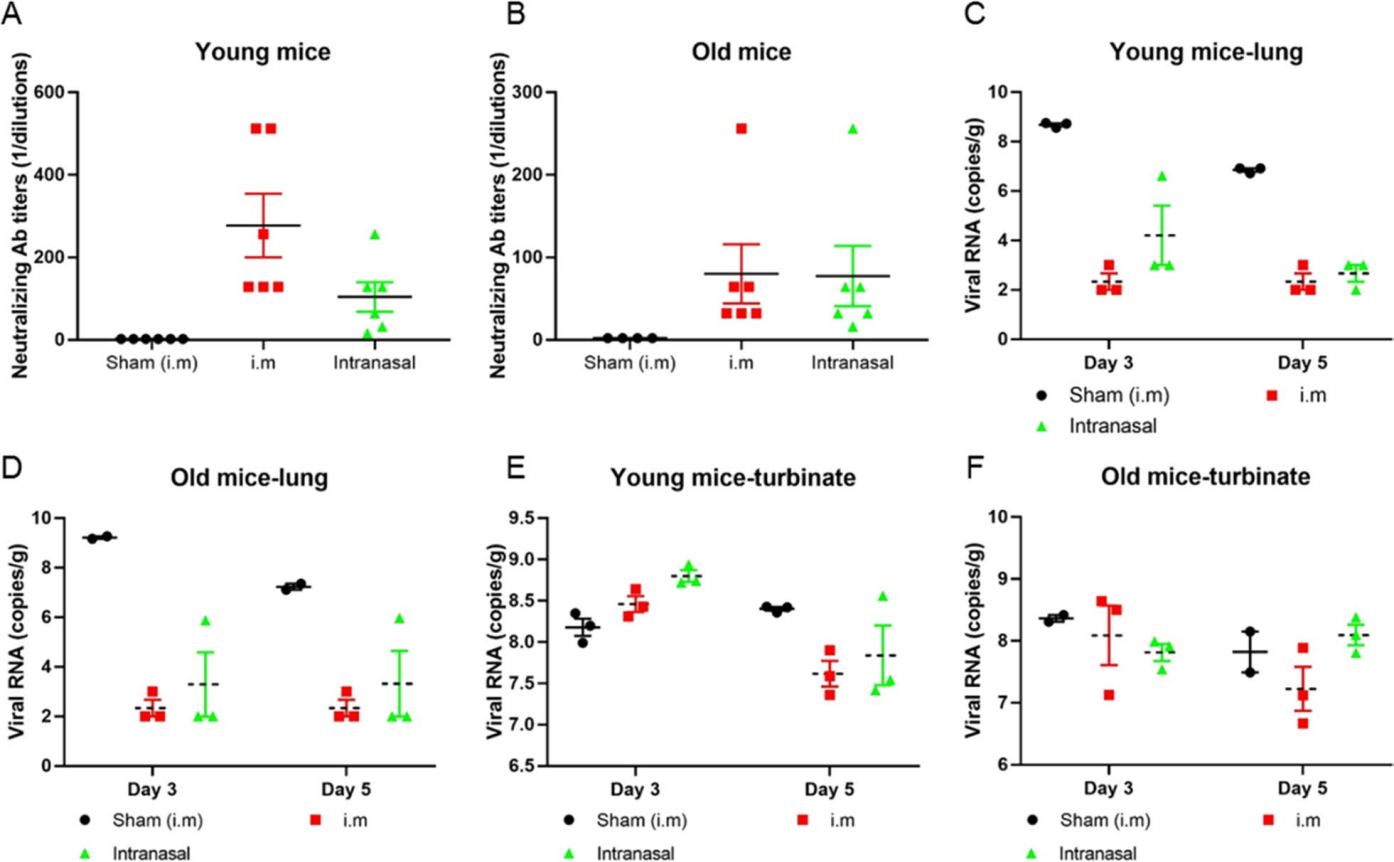
SARS-CoV-2 virus challenge test on young and old mice. **A** and **B** ssAAV5-RBD-plus significantly increased NAs in young and old BALB/c mice; **C** and **D** RBD-plus provided excellent protection in the lungs, showing a decrease in viral titre of more than 4.0 Log copies/g in lung tissues of vaccinated young and old mice; **E** and **F** After 3 and 5 days of viral inoculation, titers of infectious virus in the nose dropped nearly 1.0 Log copies/g in young and old mice.

Subsequently, a SARS-CoV-2 virus challenge test was applied to determine the protective effect of the ssAAV5-RBD-plus vaccine on 6-week old (young) and 36-week-old (old) BALB/c mice using a single dose of 6×10^10^ GCs and two routes of vaccine administration, IM and intranasal. At 40 days post-vaccination, mice were challenged intranasally with 3.6Log PFU of SARS-CoV-2 (strain HRB26M) [19]. The results showed that both administration routes of the ssAAV5-RBD-plus vaccine significantly increased NAs in young and old BALB/c mice (Fig. 3A, B). After 3 and 5 days of viral attack, viral titers were measured in the lungs and nose. As shown in Fig. 3C and D, the number of viral particles was reduced by nearly 4.0 Log copies/g in lung tissue, indicating that the ssAAV5-RBD-plus vaccine provided strong protection for the lungs, suggesting that 6×10^10^ GCs is an effective dose to induce immune protection against SARS-CoV-2. Infectious virus titers in the nose were higher than in the lungs (Fig. 3E, F). These results suggest that a single dose of the ssAAV5-RBD-plus vaccine provided substantial protection against wild-type SARS-CoV-2 in mice, in contrast to other vaccines that require multiple vaccinations[20].

### ssAAV5-RBD-plus vaccine induced long-term humoral-mediated immune and specific T-cell responses after a single-dose post-immunization

**Fig. 4:**
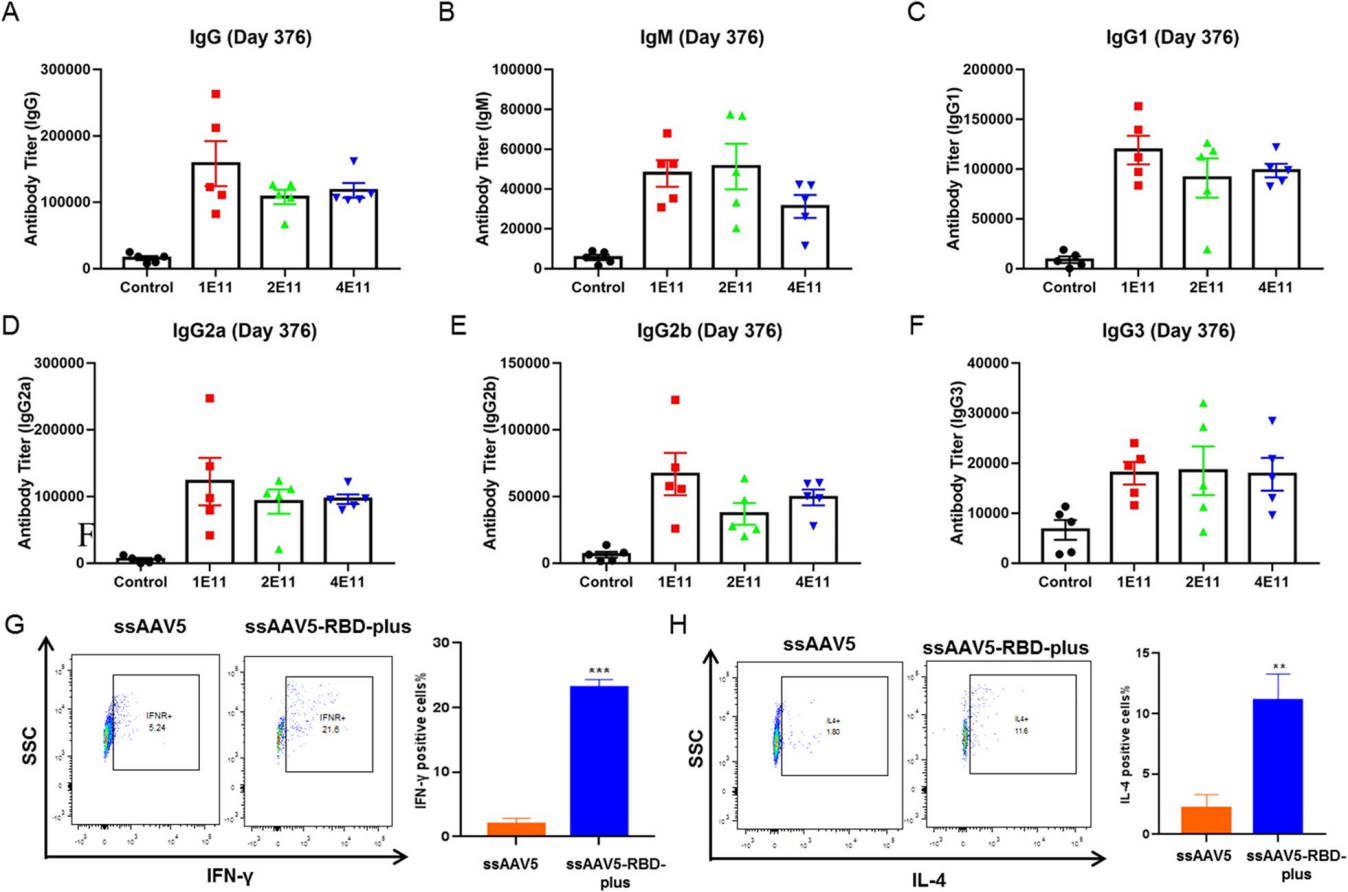
Durable immune responses and broad protection against SARS-COV-2 induced by the ssAAV5-RBD-plus vaccine in mice over one-year post-inoculation. ssAAV5-RBD-plus induced high levels of RBD-specific IgG (**A**), and prolonged and potent humoral responses, including IgM (**B**), IgG1 (**C**), IgG2a (**D**), IgG2b (**E**) and IgG3 (**F**); Secretion of IFN-γ (**G**) and IL-4 (**H**) from CD4^+^ was significantly upregulated. Values represent mean ± SEM; significant difference versus control group, **P < 0.01, ***P < 0.001.

Multiple studies have demonstrated two major requirements for vaccines to efficiently provide robust and long-lasting neutralization titers and broad protection against SARS-CoV-2 variants [21–23]. Therefore, we first investigated the long-term protective effect of the ssAAV5-RBD-plus vaccine against multiple variants over a year after immunization. Sera samples from mice were collected on day 376 post-immunization, and humoral immunity against SARS-CoV-2 was evaluated by ELISA. As shown in Fig. 4A, the ssAAV5-RBD-plus vaccine induced high levels of RBD-specific IgGs at three different doses. Meanwhile, the ssAAV5-RBD-plus vaccine produced prolonged and potent humoral responses, including primary (IgM) (Fig. 4B) and secondary antibodies (IgG subtypes) (Fig. 4C–F). We collected peripheral blood mononuclear cells (PBMCs) from mice at 376 days post-vaccination to evaluate T-cell responses. Compared to control animals, secretion of cytokines IFN-γ and IL-4 from CD4^+^ in the ssAAV5-RBD-plus group increased 4.1 and 6.5 times, respectively (Fig. 4G, H). These results indicate that the ssAAV5-RBD-plus vaccine can induce long-term specific T-cell responses after a single-dose in mice.

### **ssAAV5-RBD-plus vaccine exhibited a wide range of NAs against Alpha, Beta variants and scAAV5-RBD-plus vaccine at the concentration of 1×10**^**11**^**elicited the strongest immune response**

**Fig. 5:**
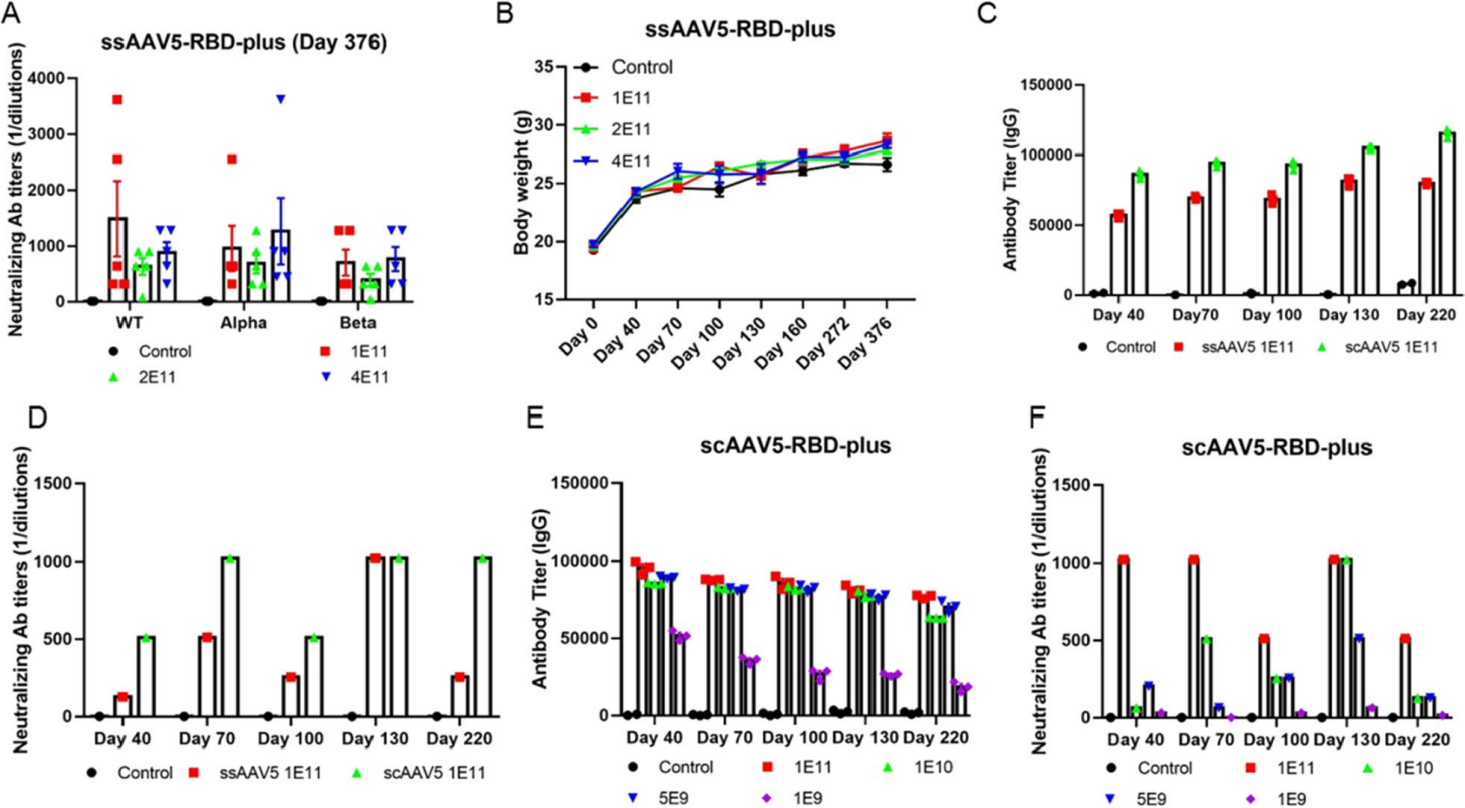
Protective effect of the ssAAV5-RBD-plus vaccine one-year post-immunization on various variants. **A** NA titers of wild-type (WT), Alpha, Beta SARS-COV-2; **B** Body weight of mice increased steadily. Immune antigenicity of ssAAV5-based and scAAV5-based vaccines in BALB/c mice; **C** IgG and **D** wild-type virus-specific NAs were evaluated at 40 days; **E** Among all doses of scAAV5-RBD-plus (1 × 10^11^, 1 × 10^10^, 5 × 10^9^ and 1 × 10^9^ GCs), 1 **×** 10^11^ GCs produced the highest IgG and NAs levels (F).

Mutations in the S protein sequence of circulating SARS-CoV-2 variants appear to reduce the efficacy of antibody neutralization induced by prior vaccination [5]. In particular, mutations in the RBD domain can impede the binding of NAs with the virus, resulting in lower efficiency of vaccines against wild-type SARS-CoV-2. Moreover, neutralizing activity declines dramatically months after infection [21, 24]. We then examined NA titers in sera from vaccinated mice against various SARS-CoV-2 variants. In wild-type mice exposed to Alpha and Beta variants, injection of the ssAAV5-RBD-plus vaccine resulted in 1:1024 NAs at a single dose of 1 × 10^11^ GCs, indicating that a single shot of ssAAV5-RBD-plus can match some currently circulating variants (Fig. 5A). Vaccination did not result in body weight loss over the 376 days post-immunization (Fig. 5B). Whole genome sequencing of tissues was performed one year after vaccination, and vector DNA sequences were not detected. Collectively, the results of our study indicate that a single dose of the ssAAV5-based RBD-plus vaccine is safe and able to induce durable immune responses in mice and produces high levels of NAs against Alpha and Beta SARS-CoV-2 variants.

AAV delivery vectors' efficiency depends on converting their single-stranded DNA (ssDNA) genome into double-stranded DNA (dsDNA). After ITR modification, self-complementary AAV vectors (scAAV) can bypass the second-strand synthesis rate-limiting step, which enhances vaccine efficiency. Therefore, we designed an scAAV5-based vaccine encoding wild-type SARS-CoV-2 RBD-plus residues 331–583 and evaluated the immunogenicity of the scAAV5-based RDB-plus vaccine in BALB/c mice. Antigen-specific humoral immune responses including IgG (Fig. 5C) and virus-specific NAs (Fig. 5D) were evaluated on days 40–220 after single-dose immunization. Compared to the ssAAV5-based vaccine, the scAAV5-based vaccine elicited higher titers of IgG and NA, indicating that the scAAV5-based vaccine elicited a more robust immune response against SARS-CoV-2. To verify the optimal dose of the scAAV-RBD-plus vaccine, we vaccinated mice by IM at doses of 1 × 10^9^, 5 × 10^9^, 1 × 10^10^ and 1 × 10^11^ GCs, respectively. The results showed that titers of IgG and NA were elevated with increasing doses, and animals vaccinated with 1 × 10^11^ GCs showed the strongest immunogenicity (Fig. 5E, F).

### scAAV5-RBD-plus vaccine not only elicited a long time humoral immunity but also provided broad immune protection against Alpha, Beta, Gamma and Delta variants

**Fig. 6:**
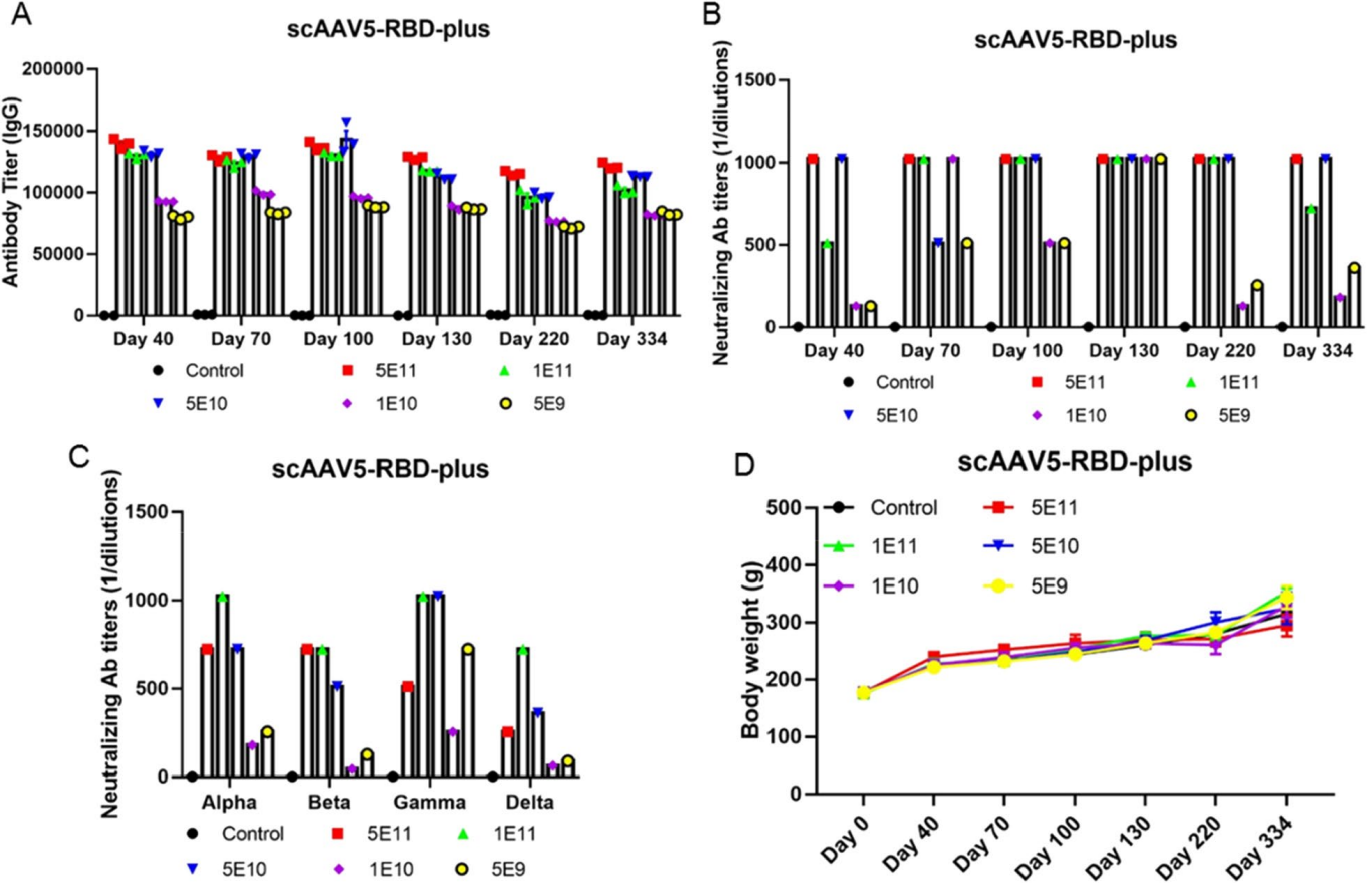
Immune antigenicity of the scAAV5-RBD-plus vaccine in Wistar rats. **A** The scAAV5-RBD-plus vaccine strongly induced IgG production in rats; **B** NAs against wild-type SARS-COV-2 remained at a high level; **C** scAAV5-RBD-plus vaccine exhibited a high titer of NAs against variants Alpha, Beta, Gamma and Delta; **D** Body weights of rats increased steadily.

To further support our findings, we tested the immune antigenicity of the scAAV-RBD-plus vaccine in Wistar rats. Sera from vaccinated rats were collected on days 40, 70, 100, 130, 220 and 334 after inoculation to evaluate the influence of humoral immunity and levels of NA. Results showed that the scAAV vaccine induced the production of IgG and wild-type virus-specific NAs throughout the 334-day experiment (Fig. 6A, B). Next, sera from all vaccinated rats were collected on day 334 and NA titers against SARS-CoV-2 variants were determined. Similar to the effect against wild-type virus, a single dose of scAAV-RBD-plus vaccine produced a high level of serum NAs against some of the major circulating variants: Alpha, Beta, Gamma and Delta (Fig. 6C). During the experimental process, vaccinated rats showed no adverse responses and exhibited a steady increase in body weight (Fig. 6D). These results indicate that the scAAV5-based RBD-plus vaccine may provide a broad and long-term immune protection against SARS-CoV-2, especially for circulating variants such as Delta.

## Discussion

This study used intramuscular delivery of rAAV5-SARS-CoV-2 vaccines in female BALB/c mice and Wistar rats models to evaluate COVID-19 vaccine candidates. It has been proved that the spike protein (S protein) of the SARS-CoV-2 virion engages the cell-surface receptor angiotensin-converting enzyme 2 (ACE2) to promote coronavirus entry into human cells. Various studies reported that the NTD, the RBD, and the S protein of SARS-CoV contain epitopes targeted by protective antibodies. In this study, we designed different rAAV5-SARS-CoV-2 vaccines expressing the RBD domain (residues 319–541), RBD-plus domain (residues 331–583), S1 protein, full-length of S protein and NTD domain to explore the immunogenicity. Compared to the classical RBD domain, the RBD-plus domain exhibited stronger IgG titers and higher neutralizing antibody titers after primary 130 days of immunization (Fig. 1). Single dose immunization of ssAAV5-RBD-plus vaccine (1 × 10^11^) not only provided both more than one year humoral and cell-mediated immune responses in mice (Figs. 2, 4) but also exhibited strong protection against wild-type SARS-CoV-2 in both old and young mice (Fig. 3).

Increasing evidence suggests that mutations in the RBD domain can induce immune escape, resulting in lower efficiency of vaccines against wild-type SARS-CoV-2. Our results shows that a single shot of ssAAV5-RBD-plus vaccine immunization (1 × 10^11^ GCs) could induce high NA titers in sera against Alpha and Beta variants (Fig. 5A, B). Most importantly, whole genome sequencing of tissues was performed one year after vaccination, and vector DNA sequences were not detected. Collectively, all our results indicates that a single shot of ssAAV5-RBD-plus produce high levels of NAs against some currently circulating variants and is safe for clinical. Meanwhile, to bypass the rate-limiting step of single-stranded AAV5 (ssAAV5), self-complementary AAV vectors (scAAV5) were designed to encode wild-type SARS-CoV-2 RBD-plus residues 331–583 and to evaluate the immunogenicity in BALB/c mice. Compared to the ssAAV5-based vaccine, the scAAV5-based vaccine elicited higher titers of IgG and NA on days 40–220 after single-dose immunization (Fig. 5C, D). We further tested the immune antigenicity of the scAAV-RBD-plus vaccine in Wistar rats. Results show that the scAAV vaccine not only induced the production of IgG and wild-type virus-specific NAs but also produced a high level of serum NAs against Alpha, Beta, Gamma and Delta variants (Fig. 6). The scAAV5-based RBD-plus vaccine may provide a broad and long-term immune protection against SARS-CoV-2, especially for circulating variants such as Delta.

In summary, the vaccines developed in the current study possess three unique advantages to combat the pandemic. First, they induced substantial and long-term immunogenicity. They maintained high titers of NAs against wild-type SARS-CoV-2 more than one year post-vaccination, without the need for a booster dose, in contrast to other vaccines [20, 25]. The RBD-plus vaccine exhibited a high level of long-lasting NAs with a range of 1:500–1:1024 responding to SARS-CoV-2 variants, including Alpha, Beta and Gamma, at an optimal dose of 1 × 10^11^ GCs, as well as the Delta variant, which is markedly resistant to currently available vaccines. Therefore, the rAAV-based vaccine represents a powerful candidate to battle the COVID-19 pandemic [10, 17]. Second, as a non-pathogenic and non-enveloped virus, the rAAV5 vector is a safe and efficient vaccine delivery vehicle. Throughout the year-long experiment, none of the vaccinated animals developed adverse effects. Furthermore, vector DNA sequence was not detected by whole genome sequencing at one-year post-vaccination. Third, rAAV vaccines are stable at − 20 °C or room temperature [17]. It would be convenient for storing and transporting of these or similar novel vaccines, contributing to their commercialization and global distribution. Taken together, our results highlight the novel rAAV5-based RBD-plus vaccine as a promising vaccine candidate to help combat the current global COVID-19 pandemic.

## Data Availability

All data generated or analysed during this study are included in this published article. Detailed data and materials are available upon e-mail request to Pro. Liang Liu (Email: lliu@gzucm.edu.cn).
